# Association of Catastrophic Health Expenditure With the Risk of Depression in Chinese Adults: Population-Based Cohort Study

**DOI:** 10.2196/42469

**Published:** 2023-08-15

**Authors:** Yaping Wang, Wannian Liang, Min Liu, Jue Liu

**Affiliations:** 1 Department of Epidemiology and Biostatistics School of Public Health Peking University Beijing China; 2 Vanke School of Public Health Tsinghua University Beijing China; 3 Institute for Healthy China Tsinghua University Beijing China; 4 Key Laboratory of Epidemiology of Major Diseases Peking University Ministry of Education Beijing China; 5 Institute for Global Health and Development Peking University Beijing China; 6 Peking University Health Science Center-Weifang Joint Research Center for Maternal and Child Health Peking University Beijing China

**Keywords:** catastrophic health expenditure, depression, universal health coverage, economic burden, socioeconomic status

## Abstract

**Background:**

Depression is one of the most common mental illnesses, and it may have a lasting effect on one’s whole life. As a form of financial hardship, catastrophic health expenditure (CHE) may be associated with depression. However, current evidence about the relationship between CHE and the risk of depression is insufficient.

**Objective:**

This study aimed to explore the relationship between CHE and the risk of depression among Chinese adults.

**Methods:**

In this study, we used 3 waves of the China Family Panel Studies (CFPS) from 2012, 2016, and 2018. The CFPS are a nationally representative study covering 25 of 31 provinces in Chinese mainland and representing nearly 94.5% of the total population. We selected eligible household heads as participants, divided them into 2 groups by CHE events at baseline (exposed group: with CHE; unexposed group: without CHE), and followed them up. Households with CHE were defined as having out-of-pocket medical expenditures exceeding 40% of the total household nonfood expenditure, and people with depression were identified by the 8-item Centre for Epidemiological Studies Depression Scale (CES-D). We first described the baseline characteristics and used logistical regression to estimate their effects on CHE events. Then, we used Cox proportional hazard models to estimate adjusted hazard ratios and 95% CIs of depression among participants with CHE compared with those without CHE. Finally, we analyzed the subgroup difference in the association between CHE and depression.

**Results:**

Of a total of 13,315 households, 9629 were eligible for analysis. Among them, 6824 (70.9%) were men. The mean age was 50.15 (SD 12.84) years. Only 987 (10.3%) participants had no medical insurance. The prevalence of CHE at baseline was 12.9% (1393/9629). Participants with a higher family economic level (adjusted odds ratio [aOR] 1.15, 95% CI 1.02-1.31) and with the highest socioeconomic development level (aOR 1.18, 95% CI 1.04-1.34) had a higher prevalence of CHE than reference groups. During a median of 71 (IQR 69-72) person-months of follow-up, the depression incidence of participants with CHE (1.41 per 1000 person-months) was higher than those without CHE (0.73 per 1000 person-months). Multivariable models revealed that the adjusted hazard ratio for the incidence of depression in participants with CHE was 1.33 (95% CI 1.08-1.64), and this association appeared to be greater in participants without outpatient services (for interaction, *P*=.048).

**Conclusions:**

CHE was significantly associated with increased risk of depression among Chinese adults. Concentrated work should be done to monitor CHE, and more efforts to ensure financial protection need to be made to prevent depression, especially for people with high health care needs.

## Introduction

There is a strong bidirectional linkage between health and poverty. To cut off this linkage, the United Nation’s Sustainable Development Goals include target 3.8, which aims to achieve universal health coverage (UHC) by 2030 [1]. UHC means that all people can receive the health services they need without experiencing financial hardship [1]. According to the World Health Organization, globally in 2017, almost 1.4 billion people experienced financial hardship due to out-of-pocket (OOP) health payments, among whom nearly 1 billion people were pushed into extreme poverty [2]. Therefore, there is still a long way to go to achieve UHC goals by 2030 considering the additional impact of COVID-19.

One of the 2 essential parts of UHC is providing financial protection for people to pay for health services [3], and the government of China has made great progress on this front. For example, as early as 2009, China reformed a series of health care reforms including three national basic medical insurance programs: (1) Urban Employee Basic Medical Insurance (UEBMI) designed for employed urban residents; (2) the Urban Resident Basic Medical Insurance (URBMI) covering the unemployed, retired, older adults, students, and children in urban areas; and (3) the New Rural Cooperative Medical Scheme (NRCMS) for rural residents [4]. Currently, China’s national basic insurance programs cover over 1.35 billion people, about 97% of the total population [5]. Additionally, to eliminate poverty, the Decision on Winning the Battle Against Poverty policy proposed by China in 2015 achieved substantial results in education, primary medical care, and the basic living needs of people living in poverty. In fact, in 2020, the government of China announced that all low-income counties in China had been lifted out of poverty [6]. Benefitting from policies and measures on financial protection of health, the incidence of catastrophic health expenditure (CHE) in China declined from 14.7% in 2010 to 8.7% in 2018 [7]. However, there are still a few people encountering financial hardship, and the impact on mental and physical health caused by CHE is still worth studying.

Depression is a prevalent mental illness globally that contributes to the global burden of diseases as the leading mental cause of mortality for all ages [8]. Once depression appears, it can have a lasting and profound influence on one’s whole life. The direct outcome of depression is a poor quality of life [9]. A study conducted on patients with schizophrenia indicated that depression has a strong negative effect on all 8 domains of subjective quality of life [10]. Furthermore, a meta-analysis showed that compared to controls, patients with depression had significant moderate cognitive deficits in executive function, memory, and attention (Cohen *d* effect sizes ranging from −0.34 to −0.65) [11]. Moreover, depression is a negative factor in cardiovascular disease (CVD) incidence, severity, and outcomes [12]. Rajan et al [13] found that depression was associated with CVD incidence (hazard ratio [HR] 1.14, 95% CI 1.05-1.24) and myocardial infarction (HR 1.14, 95% CI 1.05-1.24). Meng et al [14] also found that depression was associated with a higher risk of CVD mortality (HR 1.22, 95% CI 1.04-1.44). Therefore, the prevention and management of depression is a crucial and urgent public health issue.

A series of social, psychological, and biological factors and their complex interactions can play a role in depression occurrence [15,16]. Some study results posit that lower social support, a lower socioeconomic position, and economic difficulties are associated with a higher risk of depression [17,18], and the association is stronger between financial hardship and depression than other socioeconomic variables [19]. CHE, in theory, may have an impact on the mental health of family members because of reduced necessary expenditures. Nevertheless, current studies mostly focus on the likelihood of CHE events among people with depression, not the impact of CHE on depression occurrence [20,21]. In this study, we used 3 waves (2012, 2016, and 2018) of nationally representative data from the China Family Panel Studies (CFPS) to analyze the association of CHE with the risk of depression.

## Methods

### Study Design and Participants

Data in this study were obtained from CFPS, which is almost a nationally representative longitudinal study covering 25 of 31 provinces/municipalities in Chinese mainland (not including Xinjiang, Tibet, Inner Mongolia, Ningxia autonomous region, and Qinghai and Hainan provinces), representing nearly 94.5% of the total population in the Chinese mainland [22]. The CFPS were implemented by the Institute of Social Science Survey of Peking University to collect individual-, household-, and community-level data every 2 years. A baseline survey was conducted in 2010, and follow-up data from 2012, 2014, 2016, and 2018 were available for download from the official CFPS website [23].

**Figure 1:**
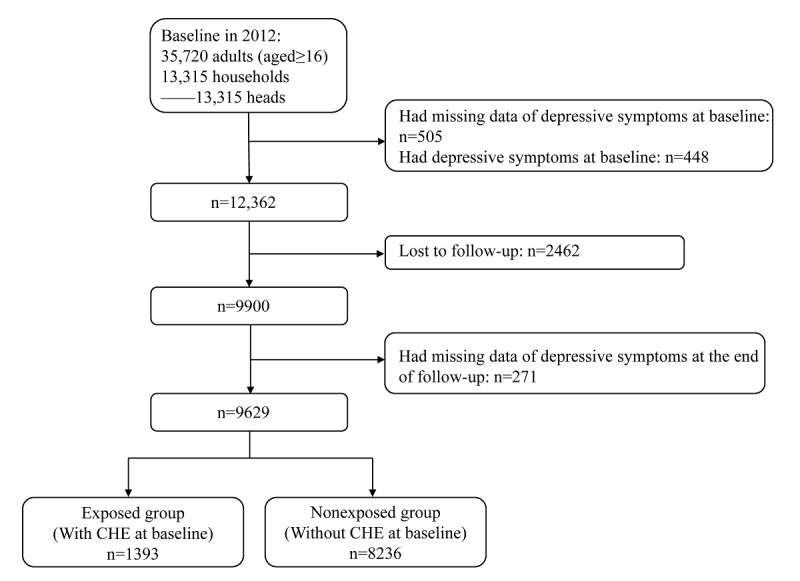
Flowchart of participants selected from China Family Panel Studies (CFPS). CHE: catastrophic health expenditure.

As the information on depression in some of the CFPS waves was deficient, we used data from 2012, 2016, and 2018. The survey in 2012 included 35,720 adults (aged ≥16 years) and 13,315 households with valid interview responses. Individuals who had missing data at baseline (n=505), had depression at baseline (n=448), were lost to follow-up (n=2462), and had missing data of depression at the end of follow-up (n=271) were excluded. Finally, a total of 9629 households (household heads) were included in this study (Figure 1).

### Ethics Approval

The CFPS, which involved human participants, were approved by the Biomedical Ethics Review Committee of Peking University (IRB00001052-14010). All written informed consent was provided by participants aged over 15 years or their parents (for those aged 15 years and under). The participants’ personal information and privacy were strictly protected by the CFPS according to the rules set by Peking University’s Biomedical Ethics Review Committee.

### Measurements of Catastrophic Health Expenditure

The measurement of catastrophic health expenditure (CHE) was based on households, where a household member was defined by marriage, blood, or adoptive relationship, as well as an ongoing economic tie [24]. The head of the household was identified as the key decision-maker when the household faced important matters and decisions. Household OOP health payments were measured as the medical expenditure of all family members excluding reimbursed expenses in the past 12 months. The yearly household food expenditure was estimated as the monthly meal expenses multiplied by 12, and the total household expenditure in the past 12 months was calculated as the sum of monthly daily expenditures (food, daily used commodities and necessities, transportation, etc) multiplied by 12 plus yearly special expenditures (electricity, medical care, clothing, etc).

Households who experienced CHE were defined as those with OOP medical expenditures exceeding 40% of the household’s capacity to pay (calculated as total household expenditure minus household food expenditure) [25]. CHE prevalence at baseline was measured as the percentage of the number of household heads incurring CHE to total participants. The formula was:


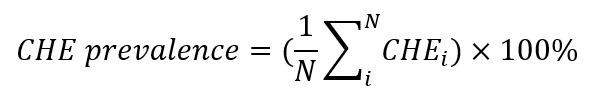


where N is the total number of participants, and *CHE_i_* is 1 when the *i*_th_ household incurred CHE and 0 otherwise.

### Measurements of Depression

Depression was measured by the 8-item Center for Epidemiological Studies Depression Scale (CES-D), which is a shortened version of the original 20-item CES-D including 8 depressive symptoms (Table S1 in Multimedia Appendix 1). CES-D was not designed as a diagnostic tool but is widely used to identify individuals at high risk of depression in general populations and various subpopulations [26]. The score of each item in the 20-item CES-D ranges from 0 to 3 according to the frequency response of depressive symptoms in the past week, with 0 indicating rarely or none of the time (<1 day), 1 indicating some or a little of the time (1-2 days), 2 indicating occasionally or a moderate amount of time (3-4 days), and 3 indicating most or all of the time (5-7 days) [26]. The commonly used 8-item CES-D asked respondents whether they experienced any of the 8 depressive symptoms “most of time during the last week” [27], which was scored according to the answer (yes=1; no=0). The 8-item CES-D used in CFPS included the same depressive symptoms but was scored by frequency response. As “most or all of the time” in frequency response is equal to the “yes” answer to “most of time last week,” we transferred the 4-score level of frequency response to the yes/no response [28]. Hence, the score of 8-item CES-D ranged from 0-8, and a total score ≥3 was deemed to indicate depression [28]. As the 8-item CES-D cannot diagnose depressive disorders, people with an 8-item CES-D score ≥3 in our study were identified as having depressive symptoms [29].

### Covariates

Covariates in this study included (1) demographic characteristics: gender (male, female), age group (16-39, 40-49, 50-49, ≥60 years), marital status (married/partnered, other), education (no or some formal education, primary school, middle school, high school and above), and insurance (without any insurance, UEBMI, URBMI, NRCMS, other); (2) health-related characteristics: self-reported health (good, medium, poor), chronic diseases (yes, no), BMI (normal, lower, overweight, obese), outpatient services (yes, no), inpatient services (yes, no), current smoking (yes, no), and drinking (yes, no); and (3) socioeconomic characteristics: residence (urban, rural), family economic level (lowest, lower, higher, highest), family size (1-2, 3-4, ≥5), and socioeconomic development level (lowest, lower, higher, highest ).

The family economic level was classified by the quartiles of household annual income, which was converted into 2012 US dollars based on purchasing power parities published by the World Bank [30] (lowest: <$4775.56, lower: $4775.56 to <$9831.46, higher: $9831.46 to <$17173.60, highest: ≥$17173.60). Numerous studies have reported that nighttime light intensity is highly associated with socioeconomic development [31,32]. Therefore, the socioeconomic development level in this study was identified by the quartiles of provinces’ mean nighttime light intensity in 2012 (lowest: <0.089, lower: 0.089-0.31, higher: 0.32-0.68, highest: ≥0.69), which was obtained from the Harvard Dataverse [33].

### Statistical Analyses

Baseline characteristics of the participants were presented as mean (SD) for continuous variables or frequencies and percentages for categorical variables. The Pearson *χ*^2^ test was used to compare distributions of CHE according to different baseline characteristics. A multivariate logistical regression model was used to analyze the determinants of CHE.

We calculated the incidence rates (number of events divided by accumulated person-month) and used the univariate and multivariate Cox proportional hazard models to estimate the HRs and 95% CIs of depression among participants with CHE compared with those without CHE. First, the model adjusted demographic characteristics in multivariate model 1, including gender, age group, marital status, education, and insurance. Next, the model adjusted health-related factors in model 2 based on model 1, including self-reported health, chronic diseases, BMI, outpatient services, inpatient services, current smoking, and current drinking. Finally, the model adjusted socioeconomic factors in model 3 based on model 2, including residence, family economic level, family size, and socioeconomic development level.

To examine the robustness of our findings, we conducted 3 sensitivity analyses. First, we defined households with CHE as those having OOP medical expenditure exceeding 25% of the total household expenditure according to the World Bank. [25] Second, we transferred the categorical variables of age group and family economic level into continuous variables and conducted the same analysis in the final model. Third, we changed the variable nighttime light intensity into the gross regional product (GRP, divided into 4 groups based on the quartiles of 2012 per capita GRP [34]) to indicate the socioeconomic development level in the final model.

Finally, the analysis was stratified by gender, age group, insurance, chronic diseases, self-reported health, outpatient services, inpatient services, residence, socioeconomic development level, and family economic level, and a multiplicative interaction term was included in the final model with the stratification variable removed for subgroup analysis.

All data were analyzed in R software (version 4.2.1; R Foundation for Statistical Computing). A 2-sided *P* value <.05 was considered to be significant.

## Results

### Baseline Characteristics

A total of 9629 participants were enrolled in this study, of which 6824 (70.9%) were male. The mean age was 50.15 (SD 12.84) years. Among the participants, 89.5% (8622/9629) were married or partnered. Only 987 (10.3%) participants had no medical insurance, and 1341 (13.9%), 603 (6.3%), and 6317 (65.6%) participants had UEBMI, URBMI, and NRCMS, respectively (Table 1).

**Table 1:** Distribution of CHE^a^ by baseline characteristics.

Characteristics				Total (N=9629)		Nonexposed group^b^ (n=8236), n (%)		Exposed group^c^ (n=1393), n (%)		Chi-square (*df*)			*P* value
**Demographic characteristics**													
	**Gender**										3.6 (1)		.06
		Male	6824		5867 (86)		957 (14)						
		Female	2805		2369 (84.5)		436 (15.5)						
	**Age group (years)**										346.3 (3)		<.001
		16-39	1931		1753 (90.8)		178 (9.2)						
		40-49	3016		2750 (91.2)		266 (8.8)						
		50-59	2320		1969 (84.9)		351 (15.1)						
		≥60	2362		1764 (74.7)		598 (25.3)						
	**Marital status**										9.1 (1)		.003
		Married/partnered	8622		7407 (85.9)		1215 (14.1)						
		Other	1007		829 (82.3)		178 (17.7)						
	**Education**										97.3 (3)		<.001
		No or some formal education	2506		2005 (80)		501 (20)						
		Primary school	2322		1980 (85.3)		342 (14.7)						
		Middle school	2789		2475 (88.7)		314 (11.3)						
		High school and above	2012		1776 (88.3)		236 (11.7)						
	**Insurance**										5.5 (4)		.24
		None	987		856 (86.7)		131 (13.3)						
		UEBMI^d^	1341		1169 (87.2)		172 (12.8)						
		URBMI^e^	603		511 (84.7)		92 (15.3)						
		NRCMS^f^	6317		5378 (85.1)		939 (14.9)						
		Other	381		322 (84.5)		59 (15.5)						
**Health-related characteristics**													
	**Self-reported health**										245.5 (2)		<.001
		Good	5874		5241 (89.2)		633 (10.8)						
		Medium	2001		1692 (84.6)		309 (15.4)						
		Poor	1754		1303 (74.3)		451 (25.7)						
	**Currently smoking**										12.6 (1)		<.001
		No	5355		4519 (84.4)		836 (15.6)						
		Yes	4274		3717 (87)		557 (13)						
	**Drinking**										16.4 (1)		<.001
		No	7282		6168 (84.7)		1114 (15.3)						
		Yes	2347		2068 (88.1)		279 (11.9)						
	**Chronic diseases**										104 (1)		<.001
		No	8268		7195 (87)		1073 (13)						
		Yes	1361		1041 (76.5)		320 (23.5)						
	**BMI**										25.8 (3)		<.001
		Normal	5596		4776 (85.3)		820 (14.7)						
		Lower	711		569 (80)		142 (20)						
		Overweight	2658		2301 (86.6)		357 (13.4)						
		Obese	664		590 (88.9)		74 (11.1)						
	**Outpatient services**										101.8 (1)		<.001
		No	7577		6624 (87.4)		953 (12.6)						
		Yes	2052		1612 (78.6)		440 (21.4)						
	**Inpatient services**										164.4 (1)		<.001
		No	8747		7644 (87.4)		1103 (12.6)						
		Yes	882		592 (67.1)		290 (32.9)						
**Socioeconomic characteristics**													
	**Residence**										11.7 (1)		<.001
		Urban	4315		3750 (86.9)		565 (13.1)						
		Rural	5314		4486 (84.4)		828 (15.6)						
	**Family economic level**										55.8 (3)		<.001
		Lowest	2988		2439 (81.6)		549 (18.4)						
		Lower	2336		2021 (86.5)		315 (13.5)						
		Higher	2366		2084 (88.1)		282 (11.9)						
		Highest	1939		1692 (87.3)		247 (12.7)						
	**Family size**										151.5 (2)		<.001
		1-2	2158		1677 (77.7)		481 (22.3)						
		3-4	4302		3833 (89.1)		469 (10.9)						
		≥5	3169		2726 (86)		443 (14)						
	**Socioeconomic development level**										6.4 (3)		.09
		Lowest	2491		2158 (86.6)		333 (13.4)						
		Lower	1492		1258 (84.3)		234 (15.7)						
		Higher	3726		3199 (85.9)		527 (14.1)						
		Highest	1920		1621 (84.4)		299 (15.6)						

^a^CHE: catastrophic health expenditure.

^b^People without CHE.

^c^People with CHE.

^d^UEBMI: Urban Employee Basic Medical Insurance.

^e^URBMI: Urban Resident Basic Medical Insurance.

^f^NRCMS: New Rural Cooperative Medical Scheme.

### CHE Prevalence

At baseline, the prevalence of CHE was 12.9% (1393/9629) among the participants. Except for gender, insurance, and socioeconomic development level, the distribution of baseline characteristics was significantly different between households with CHE and those without CHE (Table 1). The logistical regression analysis revealed that participants with poor (adjusted odds ratio [aOR] 1.64, 95% CI 1.39-1.94) and medium self-reported health (aOR 1.17, 95% CI 1-1.37), chronic diseases (aOR 1.30, 95% CI 1.11-1.52), outpatient services (aOR 1.16, 95% CI 1-1.35) and inpatient services (aOR 2.40, 95% CI 2.03-2.84), rural residence (aOR 1.21, 95% CI 1.05-1.39), family size ≥5 people (aOR 1.22, 95% CI 1.09-1.36), higher family economic level (aOR 1.15, 95% CI 1.02-1.31), and the highest socioeconomic development level (aOR 1.18, 95% CI 1.04-1.34) had a higher prevalence of CHE than reference groups (Table S2 in Multimedia Appendix 1).

### Risk of Depression

During a median of 71 (interquartile range: 69-72) person-months of follow-up, 532 (5.53%) of 9629 participants developed depression, of which 403 (75.75%) cases had no CHE and 129 (24.25%) cases had CHE. The incidence rate of depression among participants without and with CHE was 0.73 and 1.41 per 1000 person-months, respectively (Table 2). In the unadjusted analysis, participants who had CHE at baseline were associated with a 99% higher risk of depression (crude HR 1.99, 95% CI 1.63-2.42; Table 2). All multivariable–adjusted analyses showed a significant association of CHE with the risk of depression (Table S3 in Multimedia Appendix 1). In the fully adjusted model, participants with CHE had a 33% increased risk of developing depression compared to those without CHE (adjusted HR [aHR] 1.33, 95% CI 1.08-1.64; Table 2).

In the sensitivity analyses, the association between CHE and risk of depression was stable when we (1) defined CHE as OOP medical expenditure exceeding 25% of the total household expenditure, (2) transferred the categorical variables age group and family economic level to continuous variables, and (3) changed the variable nightlight time intensity into GRP to indicate socioeconomic development level in the final model (Table S4 in Multimedia Appendix 1).

**Table 2:** Association of CHE^a^ with risk of depression in the univariate and multivariate Cox proportional hazard models.

Characteristics			Outcome (N=532), n (%)		Incidence (per 1000 person-months)		Univariate model				Multivariate model^b^			
							cHR^c^ (95% CI)	*P* value		aHR^d^ (95% CI)			*P* value	
**CHE**														
	Without	403 (75.8)		0.73		Reference			Reference					
	With	129 (24.2)		1.41		1.99 (1.63-2.42)		<.001	1.33 (1.08-1.64)			.008		
**Gender**														
	Male	318 (59.8)		0.70		Reference			Reference					
	Female	214 (40.2)		1.15		1.69 (1.42-2.01)		<.001	1.51 (1.19-1.91)			.001		
**Age group (years)**														
	16-39	50 (9.4)		0.39		Reference			Reference					
	40-49	144 (27.1)		0.71		2.39 (1.93-2.97)		<.001	1.61 (1.27-2.06)			<.001		
	50-59	154 (28.9)		0.99		0.84 (0.69-1.02)		.08	0.78 (0.64-0.96)			.02		
	≥60	184 (34.6)		1.20		1.03 (0.87-1.22)		.70	0.97 (0.82-1.15)			.74		
**Marital status**														
	Married/partnered	433 (81.4)		0.75		Reference			Reference					
	Other	99 (18.6)		1.54		2.22 (1.78-2.76)		<.001	1.34 (1.05-1.70)			.02		
**Education**														
	No or some formal education	255 (47.9)		1.56		Reference			Reference					
	Primary school	121 (22.7)		0.78		0.36 (0.29-0.44)		<.001	0.63 (0.50-0.80)			<.001		
	Middle school	100 (18.8)		0.53		1.27 (1.04-1.54)		.02	1.18 (0.96-1.44)			.12		
	High school and above	56 (10.5)		0.42		0.95 (0.79-1.15)		.61	1.01 (0.83-1.22)			.95		
**Insurance**														
	None	71 (13.3)		1.11		Reference			Reference					
	UEBMI^e^	35 (6.6)		0.39		0.35 (0.23-0.52)		<.001	0.52 (0.34-0.79)			.002		
	URBMI^f^	23 (4.3)		0.57		0.51 (0.32-0.81)		.004	0.53 (0.33-0.85)			.009		
	NRCMS^g^	394 (74.1)		0.93		0.82 (0.64-1.06)		.13	0.68 (0.52-0.89)			.004		
	Other	9 (1.7)		0.35		0.30 (0.15-0.61)		.001	0.43 (0.21-0.88)			.02		
**Self-reported health**														
	Good	201 (37.8)		0.51		Reference			Reference					
	Medium	110 (20.7)		0.83		1.61 (1.28-2.03)		<.001	1.32 (1.04-1.68)			.02		
	Poor	221 (41.5)		1.93		3.89 (3.21-4.71)		<.001	2.10 (1.67-2.64)			<.001		
**Currently smoking**														
	No	314 (59.0)		0.88		Reference			Reference					
	Yes	218 (41.0)		0.77		0.86 (0.72-1.02)		.08	1.14 (0.92-1.42)			.24		
**Drinking**														
	No	435 (81.8)		0.90		Reference			Reference					
	Yes	97 (18.2)		0.62		0.67 (0.54-0.84)		.001	0.88 (0.69-1.11)			.29		
**Chronic diseases**														
	No	419 (78.8)		0.76		Reference			Reference					
	Yes	113 (21.2)		1.25		1.62 (1.31-1.99)		<.001	1.17 (0.94-1.46)			.15		
**BMI**														
	Normal	311 (58.5)		0.84		Reference			Reference					
	Lower	65 (12.2)		1.41		1.75 (1.34-2.28)		<.001	1.13 (0.86-1.48)			.40		
	Overweight	118 (22.2)		0.66		0.79 (0.64-0.98)		.03	0.92 (0.74-1.13)			.42		
	Obese	38 (7.1)		0.85		1.05 (0.75-1.47)		.78	1.13 (0.80-1.58)			.50		
**Outpatient services**														
	No	326 (61.3)		0.64		Reference			Reference					
	Yes	206 (38.7)		1.52		2.36 (1.98-2.81)		<.001	1.41 (1.16-1.72)			.001		
**Inpatient services**														
	No	477 (89.7)		0.82		Reference			Reference					
	Yes	55 (10.3)		0.95		1.18 (0.89-1.56)		.24	0.70 (0.52-0.94)			.02		
**Residence**														
	Urban	173 (32.5)		0.61		Reference			Reference					
	Rural	359 (67.5)		1.01		1.65 (1.37-1.98)		<.001	1.23 (1-1.52)			.06		
**Family economic level**														
	Lowest	240 (45.1)		1.21		Reference			Reference					
	Lower	109 (20.5)		0.70		0.55 (0.46-0.67)		<.001	0.75 (0.61-0.93)			.008		
	Higher	116 (21.8)		0.74		1.11 (0.92-1.34)		.28	1.03 (0.85-1.24)			.79		
	Highest	67 (12.6)		0.52		0.79 (0.66-0.96)		.02	0.83 (0.69-1)			.049		
**Family size**														
	1-2	165 (31)		1.17		Reference			Reference					
	3-4	200 (37.6)		0.69		0.72 (0.62-0.84)		<.001	0.96 (0.81-1.15)			.69		
	≥5	167 (31.4)		0.79		1.34 (1.16-1.55)		<.001	1.02 (0.88-1.19)			.78		
**Socioeconomic development level**														
	Lowest	174 (32.7)		1.05		Reference			Reference					
	Lower	92 (17.3)		0.93		0.69 (0.57-0.83)		.001	1.01 (0.78-1.32)			.92		
	Higher	186 (35.0)		0.75		0.92 (0.77-1.11)		.39	0.86 (0.69-1.06)			.16		
	Highest	80 (15.0)		0.63		1.01 (0.84-1.20)		.94	0.73 (0.55-0.97)			.03		

^a^CHE: catastrophic health expenditure.

^b^Adjusted for demographic characteristics (gender, age group, education, marital status, and insurance), health-related characteristics (self-reported health, smoking status, drinking, chronic disease, BMI, and outpatient and inpatient services), and socioeconomic characteristics (residence, family economic level, family size, and socioeconomic development level).

^c^cHR: crude hazard ratio.

^d^aHR: adjusted hazard ratio.

^e^UEBMI: Urban Employee Basic Medical Insurance.

^f^URBMI: Urban Resident Basic Medical Insurance.

^g^NRCMS: New Rural Cooperative Medical Scheme.

### Subgroup Analyses

**Figure 2:**
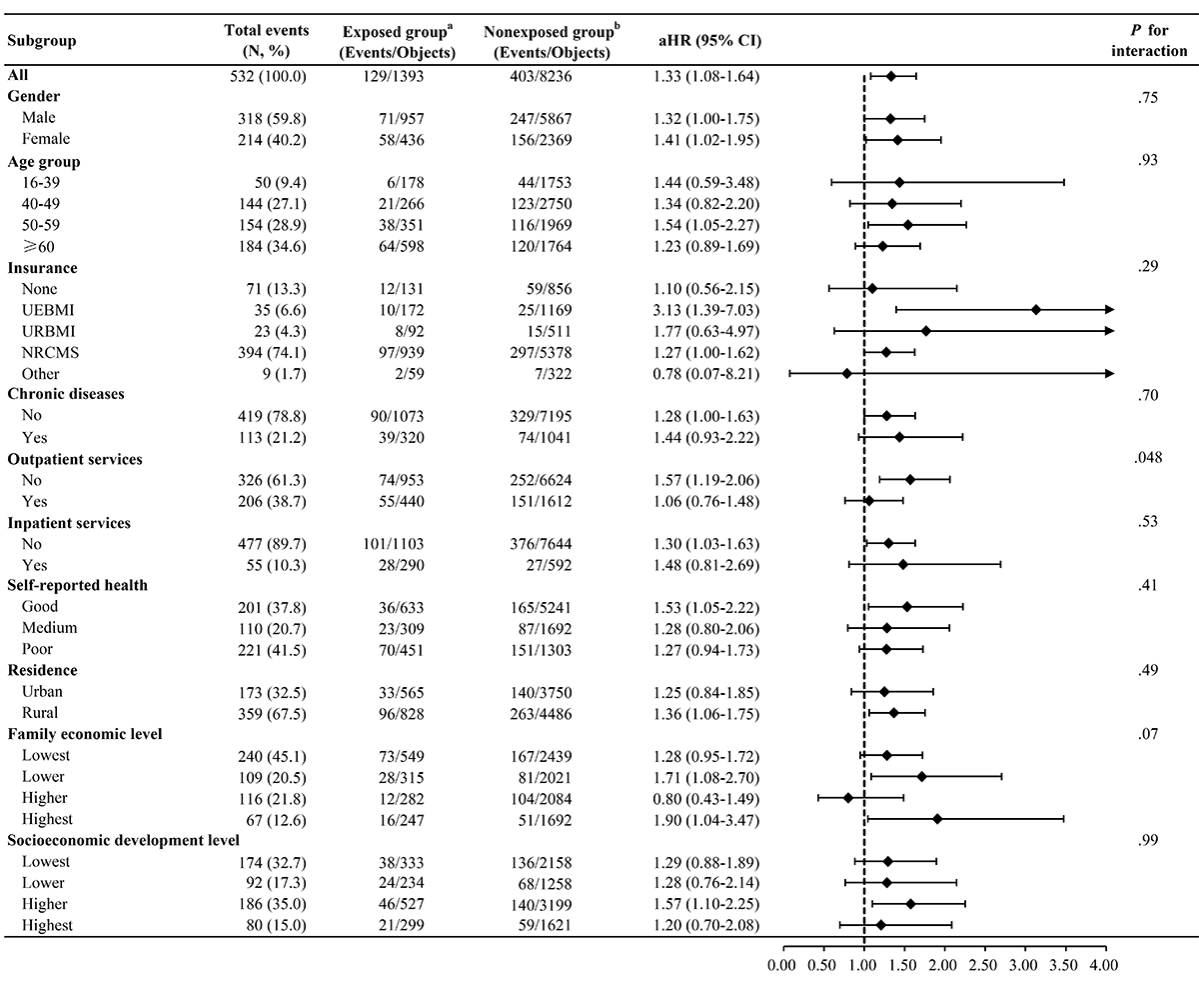
Association of catastrophic health expenditure (CHE) with risk of depression in univariate and multivariate Cox proportional hazard models. Superscripted a refers to people with CHE. Superscripted b refers to people without CHE. aHR: adjusted hazard ratio; NRCMS: New Rural Cooperative Medical Scheme; UEBMI: Urban Employee Basic Medical Insurance; URBMI: Urban Resident Basic Medical Insurance.

In the subgroup analyses, the association of CHE with the risk of depression did not appear to be modified by most selected baseline characteristics, such as gender, age group, insurance, or chronic diseases (Figure 2). However, the association between CHE and the risk of depression was found to be somewhat greater among participants without outpatient services (for interaction, *P*=.048). The risk of depression was higher among participants with CHE who did not have outpatient services (aHR 1.57, 95% CI 1.10-2.25) than those who had outpatient services (aHR 1.06, 95% CI 0.76-1.48).

## Discussion

CHE can introduce great financial pressure to a household, as household members may have to borrow money, use savings, and even sell assets [35], which may lead to a longtime negative effect on their mental health [36]. To our knowledge, this is the first cohort study on the association between CHE and the risk of depression. We found that health-related characteristics (chronic diseases, self-reported health, and outpatient and inpatient services) and socioeconomic characteristics (residence, socioeconomic development level, family size, and family economic level) had an impact on CHE. We also found that prior CHE events were strongly associated with depression experienced by household heads and that there was an interaction between CHE and outpatient services for depression.

Our results revealed that people with chronic diseases, medium and poor self-reported health, outpatient services and inpatient services, rural, family size ≥5 people, and the highest socioeconomic development level had a higher prevalence of CHE, and these findings are consistent with those of other studies [7,37]. Poor health directly leads to a higher frequency of accessing health services, further leads to a heavy financial burden of medical expenditure, and finally, leads to CHE. Nighttime light intensity is an indicator of socioeconomic development, and areas with higher economic growth may have access to diagnostic technology for most illnesses. In addition, people in areas undergoing rapid socioeconomic development may pay more attention to the early detection of diseases and other preventive health care services [38]. Consequently, health-related characteristics and socioeconomic characteristics were the main factors behind CHE.

The results of our study revealed that people with CHE had a 33% higher risk of developing depression (aHR 1.33, 95% CI 1.08-1.64) than people without CHE. Socioeconomic variables, especially financial hardship, have a great influence on depression [39]. As a form of financial hardship, CHE plays a role in depression incidence. Once a household incurred CHE, family members had to take various measures to cope with financial loss, which could take a long time to recover [35]. Given this, household heads had a higher risk of developing depression with CHE than those without CHE under financial pressure.

As CHE could trigger depression, which may further lead to adverse outcomes [40,41], it is crucial to eliminate CHE to prevent depression. The first step to eliminating CHE is based on reducing OOP health expenditures. Because poor health is associated with an increased need and use of outpatient and inpatient care, and this negative correlation can be intensified by the number of chronic diseases [37], our results suggest that relevant departments should continue to expand health promotion, especially for chronic diseases. Additionally, governments should provide sufficient and targeted financial protection, including medical insurance and poverty subsidies, to reduce OOP payments and mitigate impoverishment caused by poor health. Most studies on CHE are interested in subpopulations that have specialized diseases like cancers, HIV, and tuberculosis [42-44]. The common features of these diseases are long duration, which may even coexist with people and cause a higher risk of CHE. To decrease individual OOP health expenditure, inclined policies and measures should be introduced for these diseases. For example, to relieve the financial burden faced by people living with chronic diseases, China launched catastrophic medical insurance (critical illness insurance) in 2012 and implemented it nationwide in 2016 after city-based testing, with the aim of reimbursing patients whose OOP health expenditure exceeded a predetermined basic medical insurance level [45]. In addition, for eligible people facing unaffordable medical expenses, China provided medical assistance and even offered treatment for free or at a reduced price for some priority diseases [46]. Of course, these policies are mostly aimed at financially vulnerable people. However, our study found that people with a higher family economic level and the highest socioeconomic development level were more likely to occur CHE, which may be explained by the concentrated distribution and utilization of health services. [47] In view of this, more financial protection policies should be implemented for the whole population regardless of their economic capacity. Additionally, as the current health care delivery in China is still fragmented and treatment-based [48], with increasing incidence of age-related diseases, integrated establishment and improvement of primary health services should be intensified in health-related policies.

To cut off the linkage between CHE and depression, the second point is to halt the progression to depression. As the evidence shows in studies on financial hardship and depression, not all people experiencing financial hardship will develop depression [39]. These differing responses to CHE may be explained by various socioeconomic and psychological variables in the stress process model, such as social support, self-esteem, personal agency, and personal ability to manage difficulties [39,49,50]. Therefore, measures to prevent and regulate depression should be extended widely. Additionally, timely financial assistance should be implemented for vulnerable populations, such as people with chronic diseases and households under the minimum living guarantee.

In this study, we found that the risk of depression was higher among participants with CHE who did not have outpatient services (aHR 1.57, 95% CI 1.10-2.25) than those who had outpatient services (aHR 1.06, 95% CI 0.76-1.48). In CFPS, the variable “outpatient services” was measured by whether participants had used outpatient services in the past 2 weeks when they felt uncomfortable. Those without outpatient services included people who did not feel uncomfortable and who felt uncomfortable but did not use health services. The latter group may have a lower socioeconomic status. Moreover, heads of households with lower social support, a lower socioeconomic position, and economic difficulties were more likely to experience depression due to life pressures [17,18]. Consequently, not accessing outpatient services intensified the association between CHE and the risk of depression.

There are several limitations to our study. First, because the 8-item CES-D is not equal to clinical diagnosis, our study could only estimate the association between CHE and the risk of depressive symptoms, which calls for future research on CHE and depressive disorders. Second, due to the limitation of the original data, households with CHE did not include those facing extreme poverty who could not seek health services and whose household medical expenditure was zero. Third, expenditure data for calculating CHE were mainly based on the participants’ memory, which may be prone to recall bias.

In conclusion, people with poor health and a higher socioeconomic position had a higher prevalence of CHE and CHE was significantly associated with a higher risk of depression. To prevent depression induced by CHE, concentrated work should be made to monitor CHE, and more efforts to ensure financial protection need to be introduced and strengthened, especially for people with higher health care needs.

## Appendix

Multimedia Appendix 1 Additional tables.

## Abbreviations

aHR: adjusted hazard ratio
aOR: adjusted odds ratio
CES-D: Center for Epidemiological Studies Depression Scale
CFPS: China Family Panel Studies
CHE: catastrophic health expenditure
CVD: cardiovascular disease
GRP: gross regional product
HR: hazard ratio
NRCMS: New Rural Cooperative Medical Scheme
OOP: out-of-pocket
UEBMI: Urban Employee Basic Medical Insurance
UHC: universal health coverage
URBMI: Urban Resident Basic Medical Insurance

## Data Availability

Data from all are available to download from the official China Family Panel Studies (CFPS) website.
