# Supplementary material for: Association of Catastrophic Health Expenditure With the Risk of Depression in Chinese Adults: Population-Based Cohort Study
Source: JMIR Public Health Surveill. 2023 Aug 15;9:e42469. doi: 10.2196/42469 (PMC10466147; doi:10.2196/42469)
Supplement: Multimedia Appendix 1 [file publichealth_v9i1e42469_app1.docx]

**Association of catastrophic health expenditure with the risk of depression: a national population-based cohort study**

**Authors**: Yaping Wang^1^, Wannian Liang^2,3^, Min Liu^1^, Jue Liu^1,4,5,6^

**Author affiliations:**

1.Department of Epidemiology and Biostatistics, School of Public Health, Peking University (Yaping Wang PhD, Prof. Min Liu PhD, Prof. Jue Liu PhD)

Address: No.38, Xueyuan Road, Haidian District, Beijing 100191, China

2.Vanke School of Public Health, Tsinghua University (Prof. Wannian Liang PhD);

Address: No. 30, Shuangqing Road, Haidian District, 100084, Beijing

3.Institute for Healthy China, Tsinghua University (Prof. Wannian Liang PhD);

Address: No. 30, Shuangqing Road, Haidian District, 100084, Beijing

4. Key Laboratory of Epidemiology of Major Diseases (Peking University), Ministry of Education, Beijing, China (Prof. Min Liu PhD, Prof. Jue Liu PhD)

Address: No.38, Xueyuan Road, Haidian District, Beijing 100191, China

5. Institute for Global Health and Development, Peking University (Prof. Jue Liu PhD)

Address: No.5, Yiheyuan Road, Haidian District, Beijing, 100871, China

6. Peking University Health Science Center-Weifang Joint Research Center for Maternal and Child Health, Peking University, Beijing, China (Prof. Jue Liu PhD)

Address: No.38, Xueyuan Road, Haidian District, Beijing 100191, China

**Corresponding author:**

Prof. Min Liu, Department of Epidemiology and Biostatistics, School of Public Health, Peking University. (Tel: 86-10-8 2805146, Fax: 86-10-8 2805146, Email: liumin@bjmu.edu.cn)

Contents

[Table S1: Two versions of 8-item Center for Epidemiologic Studies Depression Scale (CES-D) 1](#_Toc135323601)

[Table S2: Multivariate logistical regression analysis of determinants of CHE 2](#_Toc135323602)

[Table S3. Association of CHE with the risk of depression in multivariable Cox proportional hazard models 4](#_Toc135323603)

[Table S4. Sensitive analyses about association between CHE and the risk of depression 5](#_Toc135323604)

# Table S1: Two versions of 8-item Center for Epidemiologic Studies Depression Scale (CES-D)

Below is a list of the ways participants might have felt or behaved during the past week.

| **Items** | **Frequency response** | **Yes/No response** |
| --- | --- | --- |
| 1. I felt depressed. | ①Rarely or none of the time (less than 1 day)  ②Some or a little of time (1-2 days)  ③Occasionally or a moderate amount of time (3-4 days)  ④Most or all of the time (5-7 days) | ①②③: No, score = 0  ④: Yes, score = 1 |
| 1. I felt that everything I did was an effort. |  |  |
| 1. My sleep was restless. |  |  |
| 1. I was happy. |  |  |
| 1. I felt lonely. |  |  |
| 1. I enjoyed life. |  |  |
| 1. I felt sad. |  |  |
| 1. I could not get “going”. |  |  |

# Table S2: Multivariate logistical regression analysis of determinants of CHE

| Characteristics | Events of CHE (n=1393, %) | aOR (95%CI) | *P* value |
| --- | --- | --- | --- |
| Demographic characteristics |  |  |  |
| Gender | 957 (68.7) |  |  |
| Male | 436 (31.3) | Reference |  |
| Female |  | 0.96 (0.82-1.13) | .65 |
| Age group | 178 (12.8) |  |  |
| 16-39 | 266 (19.1) | Reference |  |
| 40-49 | 351 (25.2) | 1.81 (1.56-2.10) | <.001 |
| 50-59 | 598 (42.9) | 1.32 (1.16-1.51) | <.001 |
| ≥60 | 178 (12.8) | 0.87 (0.77-0.99) | .03 |
| Marital status |  |  |  |
| Married/Partnered | 1215 (87.2) | Reference |  |
| Other | 178 (12.8) | 0.83 (0.69-1.01) | .07 |
| Education |  |  |  |
| No or some formal education | 501 (36.0) | Reference |  |
| Primary school | 342 (24.6) | 0.90 (0.77-1.04) | .14 |
| Middle school | 314 (22.5) | 1.13 (0.99-1.28) | .06 |
| High school and above | 236 (16.9) | 1.05 (0.93-1.18) | .47 |
| Insurance |  |  |  |
| None | 131 (9.4) | Reference |  |
| UEBMI | 172 (12.3) | 0.94 (0.72-1.23) | .65 |
| URBMI | 92 (6.6) | 1.04 (0.76-1.41) | .81 |
| NRCMS | 939 (67.4) | 1.02 (0.82-1.26) | .87 |
| Other | 59 (4.2) | 1.05 (0.73-1.51) | .78 |
| Health related characteristics |  |  |  |
| Self-reported health |  |  |  |
| Good | 633 (45.4) | Reference |  |
| Medium | 309 (22.2) | 1.17 (1.00-1.37) | .04 |
| Poor | 451 (32.4) | 1.64 (1.39-1.94) | <.001 |
| Current smoking |  |  |  |
| No | 836 (60.0) | Reference |  |
| Yes | 557 (40.0) | 0.92 (0.80-1.06) | .24 |
| Drinking |  |  |  |
| No | 1114 (80.0) | Reference |  |
| Yes | 279 (20.0) | 0.82 (0.7-0.96) | .01 |
| Chronic diseases |  |  |  |
| No | 1073 (77.0) | Reference |  |
| Yes | 320 (23.0) | 1.30 (1.11-1.52) | .001 |
| BMI |  |  |  |
| Normal | 820 (58.9) | Reference |  |
| Lower | 142 (10.2) | 1.06 (0.86-1.31) | .57 |
| Overweight | 357 (25.6) | 0.93 (0.81-1.07) | .32 |
| Obesity | 74 (5.3) | 0.71 (0.54-0.91) | .01 |
| Outpatient services |  |  |  |
| No | 953 (68.4) | Reference |  |
| Yes | 440 (31.6) | 1.16 (1-1.35) | .04 |
| Inpatient services |  |  |  |
| No | 1103 (79.2) | Reference |  |
| Yes | 290 (20.8) | 2.40 (2.03-2.84) | <.001 |
| Socioeconomic characteristics |  |  |  |
| Residence |  |  |  |
| Urban | 565 (40.6) | Reference |  |
| rural | 828 (59.4) | 1.21 (1.05-1.39) | .009 |
| Family economic level |  |  |  |
| Lowest | 549 (39.4) | Reference |  |
| Lower | 315 (22.6) | 0.84 (0.73-0.96) | .01 |
| Higher | 282 (20.2) | 1.15 (1.02-1.31) | .02 |
| Highest | 247 (17.7) | 1.06 (0.94-1.20) | .35 |
| Family size |  |  |  |
| 1-2 | 481 (34.5) | Reference |  |
| 3-4 | 469 (33.7) | 0.86 (0.76-0.97) | .01 |
| ≥5 | 443 (31.8) | 1.22 (1.09-1.36) | <.001 |
| Socioeconomic development level |  |  |  |
| Lowest | 333 (23.9) | Reference |  |
| Lower | 234 (16.8) | 1.14 (1.00-1.30) | .06 |
| Higher | 527 (37.8) | 0.93 (0.82-1.06) | .28 |
| Highest | 299 (21.5) | 1.18 (1.04-1.34) | .01 |

Notes: CHE: catastrophic health expenditure; aOR: adjusted odds ratio; CI: confident interval; UEBMI: urban employee basic medical insurance; URBMI: urban resident basic medical insurance; NRCMS: new rural cooperative medical scheme; BMI: body mass index.

# Table S3. Association of CHE with the risk of depression in multivariable Cox proportional hazard models

| CHE | Model 1^a^ | |  | Model 2^b^ | |  | Model 3^c^ | |
| --- | --- | --- | --- | --- | --- | --- | --- | --- |
|  | aHR (95%CI) | *P* value |  | aHR (95%CI) | *P* value |  | aHR (95%CI) | *P* value |
| With | Reference |  |  | Reference |  |  | Reference |  |
| Without | 1.56 (1.27-1.91) | <.001 |  | 1.36 (1.11-1.68) | .004 |  | 1.33 (1.08-1.64) | .008 |

Notes: CHE: catastrophic health expenditure; aHR: adjusted hazard ratio; CI: confident interval.

a: In model 1, hazard ratio was adjusted for demographic characteristics, including gender, age group, education, marital status and insurance.

b: Compared with model 1, hazard ratio in model 2 was additionally adjusted for health-related characteristics, including self-reported health, current smoking, drinking, chronic disease, body mass index, outpatient and inpatient services.

c: Model 3 was adjusted for all covariates, including demographic characteristics (gender, age group, education, marital status and insurance), health-related characteristics (self-reported health, smoking status, drinking, chronic disease, body mass index, outpatient and inpatient services), and socioeconomic characteristics (residence, family economic level, family size, and socioeconomic development level)

# Table S4. Sensitive analyses about association between CHE and the risk of depression

| CHE | The first sensitive analysis ^a^ | |  | The second sensitive analysis ^b^ | |  | The third sensitive analysis ^c^ | |
| --- | --- | --- | --- | --- | --- | --- | --- | --- |
|  | aHR (95%CI) | *P* value |  | aHR (95%CI) | *P* value |  | aHR (95%CI) | *P* value |
| With | Reference |  |  | Reference |  |  | Reference |  |
| Without | 1.29 (1.02-1.63) | .03 |  | 1.30 (1.05-1.60) | .01 |  | 1.32 (1.07-1.63) | .009 |

Notes: CHE: catastrophic health expenditure; aHR: adjusted hazard ratio; CI: confident interval.

a: CHE was defined as household out-of-pocket medical expenditure exceed 25% of household total expenditure.

b: The categorical variables age group and family economic level were transferred to continuous variables in the final model.

c: The variable socioeconomic development level was changed to be indicated by gross regional product level instead of nighttime light level in the final model.
